# Trend in neuraxial morphine use and postoperative analgesia after cesarean delivery in Japan from 2005 to 2020

**DOI:** 10.1038/s41598-022-22165-5

**Published:** 2022-10-14

**Authors:** Hiroshi Yonekura, Yusuke Mazda, Shohei Noguchi, Hironaka Tsunobuchi, Motomu Shimaoka

**Affiliations:** 1 Department of Anesthesiology and Pain Medicine, Fujita Health University Bantane Hospital, 3-6-10 Otoubashi, Nakagawa-ku, Nagoya, Aichi 454-8509 Japan; 2 Department of Molecular Pathobiology and Cell Adhesion Biology, Mie University Graduate School of Medicine, Mie, Japan; 3 Department of Obstetric Anesthesiology, Center for Maternal-Fetal and Neonatal Medicine, Saitama Medical Center, Saitama Medical University, Kawagoe, Japan

**Keywords:** Epidemiology, Epidemiology

## Abstract

The increasing rate of cesarean deliveries warrants obstetric anesthesiologists to deliver high-quality post-cesarean delivery analgesia. The aim of this study was to evaluate the temporal trends in the use of neuraxial morphine for cesarean deliveries and to describe the current postoperative analgesia practices. A retrospective cohort study using nationwide health insurance claims databases was conducted from 2005 to 2020 in Japan. Pregnant women who had undergone cesarean deliveries were included. The annual rate of neuraxial morphine use was extracted and analyzed. Additionally, we explored the patient- and facility-level factors associated with neuraxial morphine use through a multilevel logistic regression analysis. The cohort included 65,208 cesarean delivery cases from 2275 institutions. The prevalence of neuraxial morphine use was 16.0% (95% confidence interval [CI], 15.8–16.3) in the overall cohort. Intrathecal morphine was used in 20.6% (95% CI, 20.2–21.0) of spinal anesthesia cases. The trend in neuraxial morphine use steadily increased from 2005 to 2020. The significant predictors of neuraxial morphine use included spinal anesthesia, recent surgery, large medical facilities, and academic hospitals. Variations in the utilization of postoperative analgesia were observed. Our study described the current trend of neuraxial morphine use and the variation in postoperative analgesia practice in Japan.

## Introduction

The cesarean delivery rate is increasing in Japan (18.5% in 2013) and worldwide^1–3^. Compared to Western countries, small-scale obstetrical facilities throughout Japan provide delivery services through a decentralized delivery care system; there are 2500 medical facilities for approximately 1 million deliveries per year, and 45.5% of all deliveries are performed in small-scale private obstetric facilities with < 20 beds (clinics) managed by a few obstetricians^4,5^. According to Japan's 2020 Vital Statistics of the Ministry of Health, Labour, and Welfare, deliveries at hospitals and clinics (facilities with < 20 beds) accounted for 69% and 31% of all cesarean deliveries, respectively^5^. Despite this unique Japanese perinatal delivery care system, the maternal mortality rate in Japan is one of the lowest among the high-income countries (4 per 100,000 live births)^4,6^.

As the current strategy for post-cesarean delivery analgesia, multimodal analgesia has become the gold standard^7^. The common multimodal analgesia deploys neuraxial morphine and scheduled non-opioid analgesia, such as acetaminophen and nonsteroidal anti-inflammatory drugs (NSAIDs), to spare systemic opioids^8^. Among the multimodal analgesia components, neuraxial morphine is the most effective for post-cesarean delivery analgesia and its use is recommended by the practice guidelines because of its low cost, superior analgesic quality, and prolonged analgesic effects^9,10^. Therefore, the current epidemiology of neuraxial morphine use is important for establishing benchmarks and could provide invaluable information to help clinicians develop optimal post-cesarean delivery analgesia strategies.

To describe the real-world clinical practice of cesarean deliveries, maternal health research should focus on cases at hospitals and clinics. However, to date, the utilization of neuraxial morphine across diverse facilities has not been well studied in Japan. Thus, this study aimed to evaluate the temporal trends in the utilization of neuraxial morphine for cesarean deliveries over a 15-year period and to describe the current postoperative analgesia practice.

## Results

The initial cohort of patients who underwent cesarean delivery consisted of 77,640 eligible procedures. We excluded 12,432 cases for the following reasons: general anesthesia was used (n = 10,972) and anesthesia records were missing (n = 1460). Finally, 65,208 procedures (56,307 women) performed at 2275 institutions between January 1, 2005, and March 31, 2020, were included in the final cohort.

Table 1 summarizes the demographic and facility characteristics of the patients who received and did not receive neuraxial morphine. The patients’ mean (standard deviation [SD]) age was 33.6 (5.0) years. There were 23,862 emergency surgical cases (36.6% of the total procedures). Neuraxial morphine was used in 16.0% of the total procedures. The group that received neuraxial morphine was more likely to have a recent surgery (2015–2020) compared with the group that did not receive neuraxial morphine. The group that received neuraxial morphine was more likely to undergo cesarean deliveries in larger medical facilities, especially those with ≥ 300 beds and academic hospitals (Table 1).

**Table 1:** Patient- and facility-level characteristics of the study cohort stratified according to the use of neuraxial morphine or not.

Characteristic	All cohort (N = 65,208)	Neuraxial morphine (+) (N = 10,457)	Neuraxial morphine (−) (N = 54,751)	Absolute SMD	P value
**Age, year, mean ± SD**	33.6 ± 5.0	34.1 ± 5.0	33.5 ± 4.9	0.13	< 0.001
**Age, year**					< 0.001
< 35	38,841 (59.6)	5805 (55.5)	33,036 (60.3)	0.098	
35–39	19,635 (30.1)	3312 (31.7)	16,323 (29.8)	0.04	
40–44	6181 (9.5)	1211 (11.6)	4970 (9.1)	0.08	
> 44	551 (0.8)	129 (1.2)	422 (0.8)	0.05	
**Maternal comorbidity index score, median (IQR)**	1 (0–2)	1 (0–2)	1 (0–2)	0.15	< 0.001
**Maternal comorbidity index score**					< 0.001
0	26,435 (40.5)	3615 (34.6)	22,820 (41.7)	0.15	
1–2	30,342 (46.5)	5046 (48.3)	25,296 (46.2)	0.04	
> 2	8431 (12.9)	1796 (17.2)	6635 (12.1)	0.14	
**Charlson comorbidity index score, median (IQR)**	0 (0–0)	0 (0–0)	0 (0–0)	0.06	< 0.001
**Charlson comorbidity index**					< 0.001
< 2	63,920 (98.0)	10,179 (97.3)	53,741 (98.2)	0.06	
≥ 2	1288 (2.0)	278 (2.7)	1010 (1.8)	0.04	
**Type of surgery**					< 0.001
Elective (K898-2)	40,553 (62.2)	6143 (58.8)	34,410 (62.9)	0.08	
Emergency (K898-1)	23,862 (36.6)	4168 (39.9)	19,694 (36.0)	0.08	
Cesarean delivery with placenta previa or preterm birth (K898-3)*	793 (1.2)	146 (1.4)	647 (1.2)	0.02	
**Fiscal year**					< 0.001
2005–2009	2569 (3.9)	272 (2.6)	2297 (4.2)	0.09	
2010–2014	15,443 (23.7)	1729 (16.5)	13,714 (25.1)	0.21	
2015–2020	47,196 (72.4)	8456 (80.9)	38,740 (70.8)	0.24	
**Number of beds**					< 0.001
0–19	21,094 (32.3)	1723 (16.5)	19,371 (35.4)	0.44	
20–99	6536 (10.0)	671 (6.4)	5865 (10.7)	0.15	
100–199	2258 (3.5)	407 (3.9)	1851 (3.4)	0.03	
200–299	2278 (3.5)	484 (4.6)	1794 (3.3)	0.07	
300–499	10,549 (16.2)	2574 (24.6)	7975 (14.6)	0.26	
≥ 500	22,493 (34.5)	4598 (44.0)	17,895 (32.7)	0.23	
**Academic hospital**	7117 (10.9)	1841 (17.6)	5276 (9.6)	0.23	< 0.001

Values are given as frequencies (%) unless stated otherwise. Absolute SMD > 0.1 indicates significant imbalance between anesthesia with and without neuraxial morphine. *IQR* interquartile range, *SD* standard deviation, *SMD* standardized mean difference.

*Only available procedure code in 2010 and 2016.

Detailed descriptions of the intra/postoperative analgesic drugs used during hospitalization are shown in Tables 2 and 3. Table 2 shows the characteristics of neuraxial anesthesia use for cesarean deliveries. The group that received neuraxial morphine was more likely to receive spinal anesthesia but less likely to receive epidural anesthesia (combined spinal–epidural anesthesia [CSEA] and epidural only) and continuous epidural analgesia (22.7% vs. 38.5%) compared with the group that did not receive neuraxial morphine.

**Table 2:** Characteristics of neuraxial anesthesia used for cesarean deliveries.

Neuraxial anesthesia	All cohort (N = 65,208)	Neuraxial morphine (+) (N = 10,457)	Neuraxial morphine (−) (N = 54,751)	Absolute SMD	P value
**Type of neuraxial anesthesia**					< 0.001
Spinal anesthesia	39,948 (61.3)	8215 (78.6)	31,733 (58.0)	0.45	
CSEA	21,137 (32.4)	1775 (17.0)	19,362 (35.4)	0.43	
Epidural anesthesia	4123 (6.3)	467 (4.5)	3656 (6.7)	0.097	
**Local anesthetic for spinal anesthesia**	61,085 (93.7)	9990 (95.5)	51,095 (93.3)	0.097	< 0.001
Bupivacaine for spinal anesthesia	57,217 (87.7)	9807 (93.8)	47,410 (86.6)	0.24	< 0.001
0.5% hyper-baric	52,617	9164	43,453	0.22	< 0.001
0.5% iso-baric	4611	647	3964	0.04	< 0.001
Dibucaine	3236 (5.0)	92 (0.9)	3144 (5.7)	0.27	< 0.001
Tetracaine	670 (1.0)	92 (0.9)	578 (1.1)	0.02	0.10
**Local anesthetic for epidural anesthesia**	28,171 (43.2)	2809 (26.9)	25,362 (46.3)	0.41	< 0.001
2% Lidocaine	3839 (5.9)	553 (5.3)	3286 (6.0)	0.03	0.005
2% Mepivacaine	2851 (4.4)	233 (2.2)	2618 (4.8)	0.14	< 0.001
Ropivacaine	21,065 (32.3)	1996 (19.1)	19,069 (34.8)	0.36	< 0.001
Levobupivacaine	4641 (7.1)	333 (3.2)	4308 (7.9)	0.21	< 0.001
Bupivacaine excluding spinal anesthesia use	1497 (2.3)	170 (1.6)	1327 (2.4)	0.06	< 0.001
**Continuous infusion of local anesthetics after epidural anesthesi**a	23,462 (36.0)	2373 (22.7)	21,089 (38.5)	0.35	< 0.001

Values are given as frequencies (%) unless stated otherwise. Absolute SMD > 0.1 indicates significant imbalance between anesthesia with and without neuraxial morphine.

*CSEA* combined spinal–epidural anesthesia, *SMD* standardized mean difference.

**Table 3:** Intra/postoperative analgesics administered during hospitalization.

Analgesia	All cohort (N = 65,208)	Neuraxial morphine (+) (N = 10,457)	Neuraxial morphine (−) (N = 54,751)	Absolute SMD	P value
**Intra/postoperative analgesia administered during the hospitalization of index surgery**					
**Acetaminophen**					
Acetaminophen	23,829 (36.5)	4589 (43.9)	19,240 (35.1)	0.18	< 0.001
Acetaminophen and tramadol	285 (0.4)	230 (2.2)	55 (0.1)	0.20	< 0.001
**NSAIDs**	57,322 (87.9)	8772 (83.9)	48,550 (88.7)	0.14	< 0.001
Aspirin	9 (0.0)	1 (0.0)	8 (0.0)	0.01	1.00
Acetic derivatives	35,683 (54.7)	4249 (40.6)	31,434 (57.4)	0.34	< 0.001
Indomethacin	1701 (2.6)	30 (0.3)	1671 (3.1)	0.22	< 0.001
Diclofenac	34,107 (52.3)	4217 (40.3)	29,890 (54.6)	0.29	< 0.001
Oxicams	312 (0.5)	14 (0.1)	298 (0.5)	0.07	< 0.001
Propionates	40,708 (62.4)	6654 (63.6)	34,054 (62.2)	0.03	0.006
Ibuprofen	1065 (1.6)	167 (1.6)	898 (1.6)	0.003	0.75
Ketoprofen	791 (1.2)	8 (0.1)	783 (1.4)	0.16	< 0.001
Flurbiprofen	9394 (14.4)	1333 (12.8)	8061 (14.7)	0.06	< 0.001
Coxibs	285 (0.4)	33 (0.3)	252 (0.5)	0.02	0.040
Celecoxib	285 (0.4)	33 (0.3)	252 (0.5)	0.02	0.040
Others					
Loxoprofen	36,657 (56.2)	5983 (57.2)	30,674 (56.0)	0.02	0.025
Mefenamic acid	1258 (1.9)	234 (2.2)	1024 (1.9)	0.03	0.012
**Combined use of NSAIDs and acetaminophen**					
NSAIDs + Acetaminophen	20,551 (31.5)	3786 (36.2)	16,765 (30.6)	0.12	< 0.001
NSAIDs + Acetaminophen–opioid combination	265 (0.4)	212 (2.0)	53 (0.1)	0.19	< 0.001
Opioid*					
Buprenorphine	4034 (6.2)	94 (0.9)	3940 (7.2)	0.32	< 0.001
Fentanyl	31,122 (47.7)	7093 (67.8)	24,029 (43.9)	0.50	< 0.001
Morphine (po)	12 (0.0)	1 (0.0)	11 (0.0)	0.01	0.70
Pethidine	152 (0.2)	45 (0.4)	107 (0.2)	0.04	< 0.001
Pentazocine	25,860 (39.7)	1980 (18.9)	23,880 (43.6)	0.55	< 0.001
Tramadol (po)	274 (0.4)	95 (0.9)	179 (0.3)	0.07	< 0.001
**Miscellaneous**					
Ketamine	961 (1.5)	38 (0.4)	923 (1.7)	0.13	< 0.001
Gabapentin	1 (0.0)	0	1 (0.0)	0.01	1.00
Pregabalin	6 (0.0)	0	6 (0.0)	0.02	0.60
Naloxone	138 (0.2)	64 (0.6)	74 (0.1)	0.08	< 0.001
Metoclopramide	22,742 (34.9)	4012 (38.4)	18,730 (34.2)	0.09	< 0.001
Domperidone	137 (0.2)	47 (0.4)	90 (0.2)	0.05	< 0.001
Prochlorperazine	117 (0.2)	22 (0.2)	95 (0.2)	0.01	0.41
Droperidol	12,432 (19.1)	1830 (17.5)	10,602 (19.4)	0.05	< 0.001

Values are given as frequencies (%) unless stated otherwise. Absolute SMD > 0.1 indicates significant imbalance between anesthesia with and without neuraxial morphine.

*No use of hydrocodone, hydromorphone, methadone, oxycodone, oxymorphone, and tapentadol.

*NSAIDs* nonsteroidal anti-inflammatory drugs, *PO* per os, *SMD* standardized mean difference.

Table 3 shows the characteristics of intra/postoperative analgesic use during hospitalization. The most commonly used non-opioid analgesics for patients who had undergone cesarean deliveries were loxoprofen (56.2%), diclofenac (52.3%), and acetaminophen (36.5%). The usage proportions of acetaminophen, NSAIDs, NSAIDs plus acetaminophen, and NSAIDs plus acetaminophen–opioid combinations were 36.5%, 87.9%, 31.5%, and 0.4%, respectively. Intravenous opioids, such as pentazocine (39.7%) and buprenorphine (6.2%), were administered, whereas oral opioids, such as morphine, acetaminophen/tramadol combinations, and tramadol, were seldom prescribed (< 1%). Hydrocodone, hydromorphone, methadone, oxymorphone, oxycodone, and tapentadol were not prescribed in our cohort. The group that received neuraxial morphine was more likely to receive combined use of NSAIDs and acetaminophen (36.2% [with neuraxial morphine] vs. 30.6% [without neuraxial morphine]), acetaminophen (43.9% vs. 35.1%), acetaminophen/tramadol combination (2.2% vs. 0.1%), and fentanyl (67.8% vs. 43.9%). The group that did not receive neuraxial morphine was more likely to receive NSAIDs, specifically acetic derivatives, such as diclofenac and indomethacin, opioids (buprenorphine and pentazocine), and ketamine, compared with the group that received neuraxial morphine.

The group that received neuraxial morphine was more likely to receive naloxone and metoclopramide compared with the group that did not receive neuraxial morphine, but this difference was not a significant imbalance.

### Trends in the rate of neuraxial morphine use among women who had undergone cesarean deliveries

**Figure 1:**
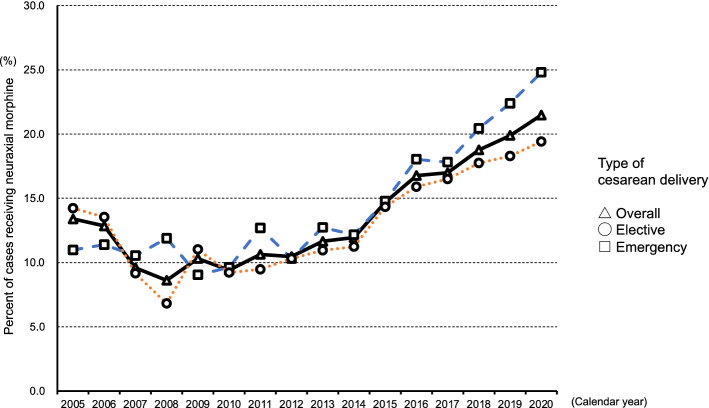
The annual rates of neuraxial morphine use and type of surgery (overall, elective, and emergency) from 2005 to 2020.

Figure 1 shows the annual rates of neuraxial morphine use and types of surgery (overall, elective, and emergency) from 2005 to 2020 according to the Japanese procedure code (the entire cohort: elective [K898-2] and emergency [code K898-1]). The procedure code K898-3, defined as cesarean delivery with placenta previa or preterm birth, was only available in 2010 and 2016; therefore, this category was not used. The rates of neuraxial morphine use of the entire cohort in 2005, 2010, and 2020 were 13.4%, 9.4%, and 21.5%, respectively. The temporal trend in neuraxial morphine use for emergency cesarean deliveries also gradually increased from 11.0% in 2005 to 24.8% in 2020. The prevalence of neuraxial morphine use was 16.0% (95% confidence interval [CI], 15.8–16.3) in the overall cohort and 17.5% (95% CI, 17.0–18.0) in emergency cases (Supplementary Table S1).

**Figure 2:**
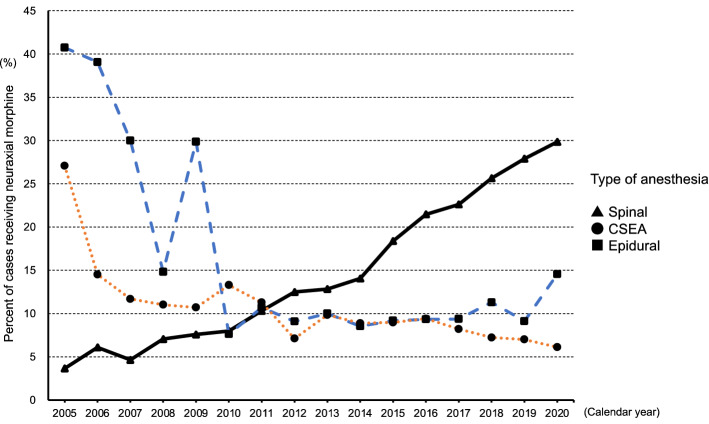
The annual rates of neuraxial morphine use and type of anesthesia (spinal, combined spinal–epidural anesthesia [CSEA], and epidural anesthesia) from 2005 to 2020.

Figure 2 shows the annual rates of neuraxial morphine use and the types of anesthesia (spinal, CSEA, and epidural anesthesia) from 2005 to 2020. The rates of neuraxial morphine use in CSEA and epidural anesthesia cases decreased with fluctuations, but those in spinal anesthesia cases steadily increased (from 3.7% in 2005 to 29.8% in 2020, P for trend < 0.001) (Supplementary Table S2).

**Figure 3:**
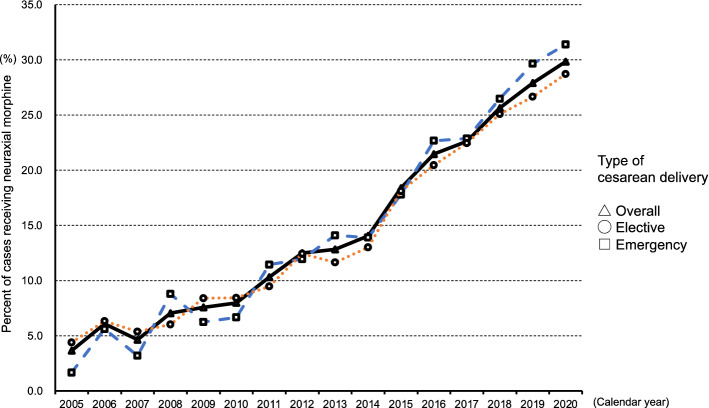
The annual rates of intrathecal morphine use in spinal anesthesia cases and type of surgery (overall, elective, and emergency) from 2005 to 2020.

Figure 3 shows the annual rates of intrathecal morphine use in spinal anesthesia cases according to the type of cesarean delivery from 2005 to 2020. The result of this sensitivity analysis was consistent with that of the main analysis (Supplementary Table S3).

### Multilevel logistic analysis of predictors associated with neuraxial morphine use in cesarean deliveries

Table 4 shows the results of the multilevel logistic regression analysis performed to examine the predictive factors associated with neuraxial morphine administration for cesarean deliveries. Significant strong predictors of neuraxial morphine use included spinal anesthesia (adjusted odds ratio [AOR], 11.26; 95% CI, 9.91–12.79) relative to CSEA, year of surgery (2015–2020) (AOR, 2.89; 95% CI, 2.27–3.69) relative to 2005–2009, number of beds (≥ 500 beds) (AOR, 12.05; 95% CI, 4.81–30.19) relative to < 500 beds, and academic hospital (AOR, 3.83; 95% CI, 1.07–13.73). Patient-level characteristics, such as maternal age, maternal comorbidity index (MCI), and Charlson comorbidity index (CCI), were not significantly associated with the use of neuraxial morphine. The area under the receiver-operating-characteristic (ROC) curve of the model was 0.69. ROC analysis using cross-validation revealed no change in the area under the receiver-operating-characteristic (AUROC) curve (mean AUROC = 0.68), indicating that the model was robust. Sensitivity analysis, which was limited to intrathecal morphine, showed results similar to those of the main analysis (Supplementary Table S4).

**Table 4:** Characteristics associated with neuraxial morphine use for cesarean deliveries.

Characteristic	Adjusted OR	95% CI	P value
**Age, year**			
< 35	Ref		
35–39	0.99	0.88–1.11	0.87
40–44	1.04	0.89–1.22	0.60
> 44	0.95	0.64–1.41	0.80
**Maternal comorbidity index score**			
0	Ref		
1–2	1.05	0.93–1.18	0.42
> 2	1.10	0.94–1.29	0.24
**Charlson comorbidity index score**			
< 2	Ref		
≥ 2	1.08	0.84–1.40	0.54
**Type of neuraxial anesthesia**			
CSEA	Ref		
Epidural anesthesia	1.89	1.43–2.48	< 0.001
Spinal anesthesia	11.26	9.91–12.79	< 0.001
**Type of surgery**			
Elective (K898-2)	Ref		
Emergency (K898-1)	0.90	0.82–0.98	0.016
Cesarean delivery with placenta previa or preterm birth (K898-3)	0.91	0.66–1.24	0.54
**Fiscal year**			
2005–2009	Ref		
2010–2014	1.03	0.80–1.33	0.80
2015–2020	2.89	2.27–3.69	< 0.001
**Number of beds**			
< 500	Ref		
≥ 500	12.05	4.81–30.19	< 0.001
**Teaching facility**			
Non-academic hospital	Ref		
Academic hospital	3.83	1.07–13.73	0.040

Institutions with less than 10 cases of cesarean delivery (956 sites; n = 3886) were excluded from the multilevel logistic regression analysis to stabilize the statistical model (1319 sites; n = 61,322). The area under the receiver-operating-characteristic curve of the model is 0.69.

*CI* confidence interval, *CSEA* combined spinal–epidural anesthesia, *OR* odds ratio.

## Discussion

In the present study, we evaluated the utilization of neuraxial morphine with temporal trends over a 15-year period and described the current postoperative analgesia practice. Our analysis revealed that the prevalence of neuraxial morphine was 16.0% in the overall cohort. Intrathecal morphine was used in 20.6% of spinal anesthesia cases. The usage rate of neuraxial morphine steadily increased from 2005 to 2020. Moreover, the significant predictors of neuraxial morphine use included spinal anesthesia, recent surgery, large medical facilities, and academic hospitals.

The increasing rate of cesarean deliveries worldwide and in Japan warrants anesthesiologists to deliver safe and high-quality anesthetic care during the perioperative period. Recently, enhanced recovery after cesarean delivery (ERAC) has been introduced and has become the standard for improving the quality of perioperative care and patient satisfaction in cesarean deliveries^7^. Among the components of ERAC, neuraxial morphine and multimodal opioid-sparing analgesia are recommended to improve the quality of care for cesarean deliveries and promote patient recovery after childbirth. However, several knowledge gaps in the current post-cesarean delivery analgesia practice still exist. First, detailed information on the current analgesia practices for cesarean deliveries in diverse nationwide facilities is limited. Therefore, a trend analysis of the use of neuraxial morphine in cesarean deliveries and a descriptive study of the current postoperative management practices are valuable for examining the quality of perioperative maternal health care. Second, patient- and facility-specific factors associated with the use of neuraxial morphine are unknown. This information could help identify barriers to standardizing the use of neuraxial morphine and improving perioperative analgesic management.

Neuraxial morphine and multimodal analgesia are the gold standards for pain management after cesarean delivery. No study has reported the exact number of women undergoing cesarean delivery who received neuraxial morphine in Japan. Our data demonstrated that, during the whole study period, neuraxial morphine was used in 16.0% of cesarean deliveries; the usage rate of neuraxial morphine, especially intrathecal morphine, gradually increased, and its administration has become the standard practice. These trends were consistent with those obtained from the United States (US) and Europe, but the rate of neuraxial opioid use in Japan was comparatively lower than those of the US (71.4% in 2008 and 83.4% in 2018^11^) and European countries (71% in Austria^12^). Our results demonstrated that recent surgeries (especially 2015–2020) were strongly associated with the utilization of neuraxial morphine. The trend in neuraxial morphine use is important in establishing benchmarks for comparison between international practices and current obstetric anesthesia practices in Japan^13^ and could provide invaluable information to help clinicians and patients in shared decision-making for post-cesarean delivery analgesia^14^.

Among the ERAC components, the use of postoperative multimodal analgesia is important to avoid inappropriate opioid use and improve maternal satisfaction. Our data demonstrated that the usage of NSAIDs plus acetaminophen and NSAIDs plus acetaminophen–opioid combination were 31.5% and 0.4%, respectively. In a cohort study conducted in the US, the usage of NSAIDs plus acetaminophen and NSAIDs plus acetaminophen–opioid combination were reported as 8.1% and 76.7%, respectively^11^. In the previous study, 81.3% of cesarean delivery cases received acetaminophen–opioid combination drugs and only 28.4% received acetaminophen^11^. Recent studies have shown that 89% of women undergoing cesarean deliveries use some form of opioid for postoperative pain, which has become a social problem, especially in the US^15^. Compared to the US cohort, our cohort was seldom prescribed acetaminophen/tramadol combinations (the only available acetaminophen–opioid combination in Japan) and tramadol (< 1%). Additionally, hydrocodone, hydromorphone, methadone, oxymorphone, oxycodone, and tapentadol were not prescribed in our cohort. This is consistent with the result of a recent survey in Japan; the usage of opioids for postoperative analgesia was reported to be substantially lower in Japan than in Western countries because of culture and strict government regulations^16^. Therefore, postoperative opioids are rarely administered in Japan. These results are highly contrasting and indicate a completely different opioid prescribing pattern compared to those in Western countries^15,17^.

The group that did not receive neuraxial morphine was more likely to receive continuous epidural analgesia, opioids (buprenorphine and pentazocine), and ketamine compared with the group that received neuraxial morphine. The group that did not receive neuraxial morphine may need long-acting analgesia, such as continuous epidural analgesia, and supplemental analgesia, such as buprenorphine, pentazocine, or ketamine, for post-cesarean delivery analgesia. Conversely, as the neuraxial morphine group used NSAIDs plus acetaminophen more frequently (36.2% [with neuraxial morphine] vs. 30.6% [without neuraxial morphine]), these results reflect the current trend in the concept of multimodal analgesia. In the US, ketorolac, diclofenac, and ibuprofen are typical NSAIDs^11^, but their use is infrequent in Japan. Instead, the essential components of multimodal post-cesarean delivery analgesia include diclofenac and loxoprofen, which are widely used during the postpartum period. As our data do not clearly distinguish between scheduling administration of NSAIDs and acetaminophen, the combined administration of NSAIDs and acetaminophen was used in only 31.5% of our cohort. A previous study in the US showed that 76.7% of patients received NSAIDs and acetaminophen–opioid combination drugs^11^. Given that the prevalence of multimodal analgesia use in Japan is very low compared to that in the US and that there is a variation in the utilization of postoperative analgesia among women undergoing cesarean deliveries, standardization of postoperative pain management after cesarean delivery is warranted to improve the quality of care and patient satisfaction.

Our results showed that neuraxial morphine use was strongly associated with facility-level characteristics, such as facility size and academic hospital status. We also showed that patient-level factors, such as age and maternal comorbidities, could not predict the use of neuraxial morphine. However, the influence of non-medical factors (fiscal year, facility size, and academic hospital status) persisted, even after adjustment, suggesting that non-medical factors are important predictors of neuraxial morphine use. The exact mechanism of this increasing trend in neuraxial morphine use cannot be explained by our data; however, in recent years, the development of subspecialty education in the field of obstetric anesthesia in large medical facilities, especially academic hospitals, may play an important role in raising awareness of the superior analgesic quality.

The strength of our study is that it is the largest nationwide study to include diverse facilities and reflects the current real-world practice to date in Japan. The JMDC database is limited to the employee-based health insured population; however, Japan has a universal health coverage system and free access to medical facilities. Hence, different types of health insurance or socioeconomic disparities would not influence our results. Thus, our results can be generalized to the majority of pregnant women undergoing cesarean deliveries in Japan^18^. Additionally, our large sample size and low rate of missing cases (1.9% [1460/77640]) can precisely estimate the patient- and facility-specific factors associated with neuraxial morphine use in clinical practice.

This study has several limitations. First, the claims-based database does not include potential confounding covariates, such as individual anesthesia/obstetric providers who are in charge of post-cesarean delivery analgesia. These confounding factors could not be accounted for in our analysis. To account for the institutional variation and clustering among institutions, we used multilevel logistic regression analysis^19^. Second, our definition of neuraxial morphine can be influenced by misclassifications, which could lead to a potential misclassification bias. To reduce misclassification bias, we defined neuraxial morphine as a morphine dose in vials of ≤ 10 mg based on a previous study^11^ because a higher morphine dose (> 10 mg) may indicate possible contamination due to intravenous administration of postoperative patient-controlled analgesia. However, as this practice is uncommon in Japan, our large sample size can mitigate this influence on our outcomes. Third, no studies to date have validated administrative claims records for the obstetric surgical population. The determination of comorbidity statuses, procedures, and medication used in the study depended on the accuracy of administrative claims records. The records of the diagnoses and procedures in the Japanese administrative data were validated with relatively moderate sensitivity, high specificity, and high positive predictive values (PPV)^20,21^. By limiting the study population to patients with less common diseases (e.g., the obstetric population), the PPV would be lower, and thus it remains unclear whether the results of the validation study can be extrapolated to our population. Regarding medication claims, the medication records are linked to the payment system for reimbursement of drugs. Additionally, morphine is a narcotic and requires a separate prescription in Japan, and thus opioid medications are strictly recorded and reimbursed. Therefore, it is clinically reasonable to infer a low possibility of coding errors or incomplete medication claims records. As data quality and accuracy are critical factors for real-world data analysis, a future study is required to investigate the validity of claims-based medication use. Considering the scarcity of evidence regarding current trends of post-cesarean delivery analgesia, the results of this study can provide the best available real-world evidence to date.

Another limitation is the time span of our dataset. As the data were collected between 2005 and 2020, the trend in analgesic use or available drugs has changed; therefore, it is uncertain to what degree the rates of postoperative analgesia use have changed since 2005. However, given the surprising lack of national and international data on the rates of neuraxial morphine use for cesarean deliveries, our data are important for setting local benchmarks and treatment goals. In addition, our findings may guide future studies to further delineate practice patterns by facility and potentially improve the quality of care for cesarean deliveries and promote patient recovery after childbirth. Finally, the perinatal care system and obstetric anesthesia practices in Japan are completely different from those of Western countries. A previous government survey found that 31% of cesarean deliveries were performed in small facilities (< 20 beds)^5^. Compared to other Western countries, the Japanese health care system has an insufficient functional differentiation between hospitals and clinics^18,22^.

In conclusion, our data could be useful for identifying the current trend in neuraxial morphine administration and the variation in postoperative analgesia practice in Japan for over 15 years. Moreover, our data provide valuable information for the standardization of post-cesarean delivery analgesia practice, as we compared national and global data.

## Methods

This study was approved by the Institutional Review Board of Fujita Health University and Mie University (HM21-369 and H2019-167, respectively), which waived the requirement for obtaining additional informed consent from the participants owing to the anonymous nature of the data. This study was conducted in accordance with the Declaration of Helsinki and followed the Strengthening the Reporting of Observational Studies in Epidemiology (STROBE) statement^23^.

### Data source

We used the nationwide health insurance claims database obtained from the medical database vendor JMDC Co., Ltd (Tokyo, Japan)^24^. This database collects anonymized inpatient, outpatient, and dispensing claims data from various health insurance associations and has accumulated over 10 million people since 2005 (approximately 10% of the Japanese population)^25^. As this database is sourced from health insurance associations for company employees and their families, it is representative of the relatively young generation, which is advantageous for maternal health care research^26,27^. This database included data on patient demographics, inpatient and outpatient claims (diagnosis, procedure codes, and medication information), and facility characteristics. Clinical diagnoses were coded according to the International Classification of Diseases 10th revision (ICD-10), and the procedure code was defined using the Japanese standardized procedure codes (K codes). The medications administered were date-stamped using the World Health Organization Anatomical Therapeutic Chemical (WHO-ATC) classification system. Details of the JMDC database and included variables have been described in previous studies^13,18,24^. The distribution of demographics (age and sex) and claims code provided by each chapter of ICD-10 in the JMDC database are almost consistent with the governmental statistics, which were aggregates of the national database for the total population of Japan health insurance claims and specific health checkups^28^. The records of the diagnoses and procedures in the Japanese administrative data were validated with relatively moderate sensitivity and high specificity^20,21^. Previous studies using the JMDC database reported the validity of diagnostic and medication code-based algorithms for common chronic conditions such as hypertension and diabetes. The sensitivity and specificity of claims-based algorithms for common chronic conditions were > 75% and > 90%, respectively^21^. Data from the JMDC database have been used in multiple pharmacoepidemiological studies that have been published in peer-reviewed journals^27,29,30^.

### Study cohort

We included pregnant women who underwent elective or emergency cesarean deliveries identified by the Japanese procedure K codes (K898-1, K898-2, and K898-3)^31^, which were performed under neuraxial anesthesia between January 1, 2005, and March 31, 2020. We used a combination of Japanese L codes (anesthesia-related codes) and WHO-ATC drug claims to categorize neuraxial anesthesia as spinal anesthesia, CSEA, or epidural anesthesia (Supplementary Table S5). Cases with unclear information about the type of anesthesia or those receiving general anesthesia were excluded.

### Patient- and facility-level variables

The patient characteristics examined included age, MCI, and CCI. We used the MCI developed by Bateman et al.^32^ and the modified CCI by Quan et al.^33^ using the ICD-10 code algorithm to ascertain the burden of systemic comorbidities. The MCI is a validated comorbidity score in obstetric populations designed to assess maternal comorbidity^34,35^; the MCI scores were calculated based on 20 primary diseases and maternal age > 35 years. The CCI is also a validated comorbidity score index. Higher MCI and CCI scores indicate a high burden of systemic disease. We categorized the MCI score as 0, 1–2, or > 2, and the CCI score as < 2 and > 2 based on previous studies^36^. The medical facility size was categorized in the JMDC database as follows: 0–19, 20–99, 100–199, 200–299, 300–499, or ≥ 500 beds. Hospital characteristics were simplified into the following two categories: academic hospitals (university hospitals and public hospitals with advanced functions) and non-academic hospitals^18^. We categorized hospital sizes as < 500 or ≥ 500 beds.

### Outcomes

The primary outcome was the proportion of patients who received neuraxial morphine for cesarean delivery. The secondary outcome was the analgesic drugs administered during hospitalization after cesarean delivery (Supplementary Table S5). The administration routes (neuraxial, intravenous, oral, etc.) and concentration of each analgesic were determined based on the text information of their Japanese brand name drugs. In general, neuraxial morphine is used within a dose of ≤ 10 mg; thus, we defined “neuraxial morphine” as a combination of neuraxial anesthesia code and morphine drug information (ATC code: N02AA01) in vials with a dose of ≤ 10 mg^11^. In the sensitivity analysis for testing the robustness of our results, we limited the neuraxial morphine to intrathecal morphine in spinal anesthesia cases (also known as “single-shot spinal” anesthesia) to exclude the combined use of epidural anesthesia. We described the utilization of antiemetics and opioid reversal agents as a proxy for the side effects of nausea/vomiting and respiratory depression due to morphine, respectively.

### Statistical analysis

We calculated the proportions of neuraxial morphine use in the overall cohort and patient groups stratified by the type of cesarean delivery (overall, elective, or emergency) and the type of anesthesia (spinal, CSEA, or epidural). Background characteristics and intra/postoperative analgesia management were assessed in anesthesia cases with and without neuraxial morphine. Continuous variables are presented as the mean (SD) or median (interquartile range [IQR]), and categorical variables as the number (proportion). In general, the larger the sample size, the smaller the P value obtained from the baseline comparison. Therefore, we calculated the standardized mean differences (SMDs) in addition to P values when comparing cases receiving neuraxial anesthesia with and without neuraxial morphine; an absolute SMD > 0.1 indicates a meaningful imbalance between group differences^37^. Differences between groups were tested using the Pearson χ^2^ test (Fisher’s exact test) and Student’s *t* test (Mann–Whitney *U* test) for categorical and continuous variables, respectively, as appropriate. To describe the trend in neuraxial morphine use, the rate of women who received neuraxial morphine was calculated each year over the 15-year study period. The trend in proportions was assessed using the Cochrane-Armitage trend test^38^. To account for the variation of institutional practice and clustering among institutions, we performed a multilevel logistic regression analysis^19^. The GLIMMIX procedure in SAS 9.4 (SAS Institute Inc., Cary, NC, USA) was used. Institutions with < 10 cesarean deliveries were excluded from the regression analysis to stabilize the statistical model. We included the institution as a random effect and the following covariates as fixed effects: maternal age category, MCI category, CCI category, type of anesthesia, type of surgery, year of surgery, facility size category, and academic hospital status. The adjusted odds ratios and 95% CI for fixed effects were reported along with the P values. The model was validated internally using fivefold cross-validation. In the sensitivity analysis, we only analyzed spinal anesthesia cases receiving neuraxial morphine and performed descriptive analyses and multilevel logistic regression analyses. All analyses were performed using SAS 9.4, and a two-sided α level of 0.05 was considered statistically significant.

## Supplementary Information

Supplementary Information.

## Data Availability

The datasets analyzed in this study are available from the corresponding author upon reasonable request.
