# Supplementary material for: Trend in neuraxial morphine use and postoperative analgesia after cesarean delivery in Japan from 2005 to 2020
Source: Sci Rep. 2022 Oct 14;12:17234. doi: 10.1038/s41598-022-22165-5 (PMC9568599; doi:10.1038/s41598-022-22165-5)
Supplement: Supplementary file 1 — Supplementary Information. [file 41598_2022_22165_MOESM1_ESM.pdf]

Supplemental Digital Content

Title: Trend in neuraxial morphine use and postoperative analgesia after cesarean delivery in Japan from 2005 to 2020.

Hiroshi Yonekura[1,2,*] (MD, MPH), Yusuke Mazda[3] (MD, PhD), Shohei Noguchi[3] (MD, PhD), Hironaka Tsunobuchi[1] (MD, PhD), Motomu Shimaoka[2] (MD, PhD)

[1] Department of Anesthesiology and Pain Medicine, Fujita Health University Bantane Hospital, Aichi, Japan

[2]Department of Molecular Pathobiology and Cell Adhesion Biology, Mie University Graduate School of Medicine, Mie, Japan

[3]Department of Obstetric Anesthesiology, Center for Maternal-Fetal and Neonatal Medicine, Saitama Medical Center, Saitama Medical University, Kawagoe, Japan

Supplemental Legends

Supplemental Table S1. Trend in neuraxial morphine administration by cesarean delivery type from 2005 to 2020.

Supplemental Table S2. Trend in neuraxial morphine administration by type of anesthesia from 2005 to 2020.

Supplemental Table S3. Trend in intrathecal morphine administration in spinal anesthesia cases by type of cesarean delivery from 2005 to 2020.

Supplemental Table S4. Characteristics associated with intrathecal spinal morphine administration in spinal anesthesia for cesarean deliveries.

Supplemental Table S5. List of all the claims and drug codes used to identify the type of anesthesia and analgesics administered during hospitalization.

Table S1. Trend in neuraxial morphine administration by cesarean delivery type from 2005 to 2020

Fiscal year	Type of cesarean deliveries, % (95% CI)		
	Overall (N=65,208)	Elective (N=40,553)	Emergency (N=23,862)
2005	13.4 (9.7–17.1)	14.2 (9.8–18.7)	11.0 (4.2–17.7)
2006	12.9 (9.5–16.2)	13.5 (9.4–17.6)	11.4 (5.8–17.0)
2007	9.6 (6.8–12.4)	9.2 (5.8–12.5)	10.5 (5.3–15.7)
2008	8.6 (6.3–10.9)	6.8 (4.2–9.4)	11.9 (7.4–16.3)
2009	10.3 (8.3–12.3)	11.0 (8.4–13.6)	9.0 (5.8–12.2)
2010	9.4 (7.9–10.9)	9.2 (7.4–11.1)	9.6 (7.0–12.3)
2011	10.6 (9.3–12.0)	9.5 (7.8–11.1)	12.7 (10.2–15.2)
2012	10.5 (9.3–11.6)	10.3 (8.9–11.8)	10.3 (8.4–12.2)
2013	11.7 (10.7–12.6)	10.9 (9.8–12.1)	12.7 (11.1–14.4)
2014	12.0 (11.0–12.9)	11.2 (10.1–12.4)	12.2 (10.6–13.7)
2015	14.7 (13.8–15.6)	14.3 (13.2–15.4)	14.8 (13.3–16.2)

2016	16.8 (15.9–17.6)	15.9 (14.9–16.9)	18.0 (16.6–19.4)
2017	17.0 (16.2–17.8)	16.5 (15.5–17.5)	17.8 (16.5–19.1)
2018	18.8 (18.0–19.5)	17.7 (16.8–18.6)	20.4 (19.2–21.7)
2019	19.9 (19.1–20.6)	18.3 (17.3–19.2)	22.4 (21.1–23.7)
2020	21.5 (19.8–23.2)	19.4 (17.3–21.5)	24.8 (21.9–27.7)
Total	16.0 (15.8–16.3)	15.1 (14.8–15.5)	17.5 (17.0–18.0)

CI, confidence interval

Table S2. Trend in neuraxial morphine administration by the type of anesthesia from 2005 to 2020

Fiscal year	Type of anesthesia, % (95% CI)		
	Spinal anesthesia (N=39,948)	CSEA (N=21,137)	Epidural anesthesia (N=4,123)
2005	3.7 (1.2–6.1)	27.1 (14.5–39.7)	40.7 (27.6–53.9)
2006	6.1 (3.2–9.0)	14.5 (5.8–23.3)	39.1 (27.1–51.0)
2007	4.6 (2.2–7.1)	11.7 (4.5–18.9)	30.0 (18.4–41.6)
2008	7.1 (4.5–9.6)	11.0 (5.4–16.7)	14.8 (5.3–24.3)
2009	7.6 (5.4–9.7)	10.7 (6.6–14.8)	29.9 (19.7–40.1)
2010	8.0 (6.3–9.7)	13.3 (10.0–16.6)	7.6 (2.5–12.7)
2011	10.3 (8.6–12.0)	11.3 (8.7–13.9)	10.7 (5.4–16.0)
2012	12.5 (10.9–14.1)	7.1 (5.4–8.8)	9.1 (5.2–13.0)
2013	12.8 (11.6–14.1)	9.8 (8.3–11.4)	10.0 (6.6–13.5)
2014	14.1 (12.8–15.3)	8.9 (7.5–10.2)	8.5 (5.3–11.7)
2015	18.4 (17.2–19.6)	9.0 (7.7–10.2)	9.2 (6.4–11.9)

2016	21.5 (20.3–22.6)	9.4 (8.3–10.5)	9.3 (6.8–11.8)
2017	22.6 (21.5–23.7)	8.2 (7.2–9.2)	9.4 (7.0–11.8)
2018	25.6 (24.6–26.7)	7.2 (6.4–8.1)	11.3 (8.8–13.8)
2019	27.9 (26.8–29.0)	7.0 (6.2–7.8)	9.1 (6.8–11.5)
2020	29.8 (27.4–32.3)	6.1 (4.3–7.9)	14.5 (8.0–21.1)
Total	20.6 (20.2–21.0)	8.4 (8.0–8.8)	11.3 (10.4–12.3)

CI, confidence interval; CSEA, combined spinal-epidural anesthesia

Table S3. Trend in intrathecal morphine administration in spinal anesthesia cases by the type of cesarean delivery from 2005 to 2020

Fiscal year	Type of cesarean deliveries, % (95% CI)		
	Overall (N=39,948)	Elective (N=23,901)	Emergency (N=15,648)
2005	3.7 (1.2–6.1)	4.4 (1.2–7.6)	1.7 (0.0–4.9)
2006	6.1 (3.2–9.0)	6.3 (2.7–9.9)	5.6 (0.8–10.4)
2007	4.6 (2.2–7.1)	5.4 (2.1–8.6)	3.2 (0.0–6.7)
2008	7.1 (4.5–9.6)	6.0 (3.1–9.0)	8.8 (4.2–13.3)
2009	7.6 (5.4–9.7)	8.4 (5.5–11.3)	6.3 (3.1–9.4)
2010	8.0 (6.3–9.7)	8.4 (6.2–10.7)	6.7 (4.0–9.4)
2011	10.3 (8.6–12.0)	9.5 (7.4–11.6)	11.4 (8.6–14.4)
2012	12.5 (10.9–14.1)	12.4 (10.4–14.5)	11.9 (9.4–14.5)
2013	12.8 (11.6–14.1)	11.6 (10.1–13.2)	14.1 (11.9–16.3)
2014	14.1 (12.8–15.3)	13.0 (11.4–14.6)	13.9 (11.8–16.0)
2015	18.4 (17.2–19.6)	18.1 (16.5–19.7)	17.8 (15.8–19.8)

2016	21.5 (20.3–22.6)	20.5 (19.0–21.9)	22.7 (20.8–24.6)
2017	22.6 (21.5–23.7)	22.5 (21.0–23.9)	22.9 (21.1–24.6)
2018	25.6 (24.6–26.7)	25.1 (23.7–26.4)	26.5 (24.8–28.1)
2019	27.9 (26.8–29.0)	26.7 (25.3–28.1)	29.6 (27.9–31.4)
2020	29.8 (27.4–32.3)	28.7 (25.6–31.9)	31.4 (27.6–35.2)
Total	20.6 (20.2–21.0)	19.8 (19.3–20.3)	21.5 (20.9–22.2)

CI, confidence interval

Table S4. Characteristics associated with intrathecal morphine administration in spinal anesthesia for cesarean deliveries

Characteristic	Adjusted OR	95% CI	P value
Age, year			
<35	ref		
35-39	1.03	0.88–1.21	0.70
40-44	1.03	0.83–1.27	0.82
>44	0.98	0.58–1.67	0.94
Maternal comorbidity index score			
0	ref		
1–2	0.99	0.84–1.16	0.86
>2	1.05	0.85–1.31	0.65

Charlson comorbidity index score			
<2	ref		
≥2	1.08	0.75–1.54	0.68
Type of surgery			
Elective (K898-2)	ref		
Emergency (K898-1)	0.77	0.68–0.87	<.001
Cesarean delivery with placenta previa or preterm birth (K898-3)	0.96	0.64–1.45	0.85
Fiscal year			
2005–2009	ref		
2010–2014	2.99	2.04–4.38	<.001
2015–2020	15.09	10.36–21.98	<.001
Number of beds			

<500	ref		
≥500	14.85	3.83–57.57	<.001
Teaching facility			
Non-academic hospital	ref		
Academic hospital	14.31	1.85–110.93	0.011

CI, confidence interval; CSEA, combined spinal–epidural anesthesia; OR, odds ratio.

Table S5. List of all the claims and drug codes used to identify the type of anesthesia and analgesics administered during hospitalization

Anesthesia information coded according to the Japanese claims classification (anesthesia category: L)	
General anesthesia	L008
Spinal anesthesia	L004
Epidural anesthesia	L002
Continuous infusion of local anesthetic after epidural anesthesia (per day) (excluding the day of anesthesia)	L003
Drug prescription information coded according to the World Health Organization Anatomical Therapeutic Chemical (WHO-ATC) classification	
Local anesthetic for neuraxial anesthesia	
Bupivacaine	N01BB01
Tetracaine	N01BA03
Cinchocaine	N01BB06, N01BB20
Lidocaine	N01BB02
Mepivacaine	N01BB03
Ropivacaine	N01BB09
Levobupivacaine	N01BB10
Lidocaine, combinations	N01BB52
Analgesics	

Acetaminophen	N02BE01
Tramadol and acetaminophen	N02AJ13
NSAIDs	
Aspirin	N02BA01
Acetic derivatives	M01AB
Indomethacin	M01AB01
Diclofenac	M01AB05
Oxicams	M01AC
Propionates	M01AE
Ibuprofen	M01AE01
Ketoprofen	M01AE03
Flurbiprofen	M01AE09
Coxibs	M01AH
Celecoxib	M01AH01
Others	M01AX
Loxoprofen	M02AA31
Mefenamic acid	M01AG01
Opioid	
Buprenorphine	N02AE01
Fentanyl	N01AH01
Morphine	N02AA01

Pethidine	N02AB02
Pentazocine	N02AD01
Tramadol	N02AX02
Miscellaneous	
Ketamine	N01AX03
Gabapentin	N03AX12
Pregabalin	N03AX16
Naloxone	V03AB15
Metoclopramide	A03FA01
Domperidone	A03FA03
Prochlorperazine	N05AB04
Droperidol	N05AD08

*In this study, we identified the route and concentration of drugs by text information.
